# Supplementary material for: A high-resolution mRNA expression time course of embryonic development in zebrafish
Source: eLife. 2017 Nov 16;6:e30860. doi: 10.7554/eLife.30860 (PMC5690287; doi:10.7554/eLife.30860)
Supplement: Supplementary file 6. [file elife-30860-supp6.zip › biolayout-clusters-files/Cluster107-genes.html]

Cluster107


# Cluster107: Genes

| Ensembl ID | Gene Name | Chr | Start | End | Biotype |
| --- | --- | --- | --- | --- | --- |
| ENSDARG00000102315 | RAI2 | KN150079.1 | 12700 | 14654 | protein\_coding |
| ENSDARG00000021833 | ahr2 | 22 | 13021493 | 13140409 | protein\_coding |
| ENSDARG00000043257 | ckbb | 20 | 20917074 | 20922116 | protein\_coding |
| ENSDARG00000032831 | htra1a | 17 | 21899295 | 21939720 | protein\_coding |
| ENSDARG00000015312 | klf12a | 1 | 33764287 | 33824155 | protein\_coding |
| ENSDARG00000070080 | nbeaa | 10 | 34482439 | 34791137 | protein\_coding |
| ENSDARG00000020794 | neurod6b | 2 | 50892279 | 50894921 | protein\_coding |
| ENSDARG00000003877 | pbrm1 | 11 | 3890369 | 3940502 | protein\_coding |
| ENSDARG00000054191 | pgk1 | 21 | 37761018 | 37776965 | protein\_coding |
| ENSDARG00000104891 | si:dkey-109j17.5 | 23 | 19276293 | 19280076 | protein\_coding |
| ENSDARG00000104244 | twsg1a | 2 | 55065750 | 55100113 | protein\_coding |
| ENSDARG00000089877 | wu:fj40g07 | 7 | 24215083 | 24220727 | protein\_coding |
